# Human recombinant arginase I [HuArgI (Co)-PEG5000]-induced arginine depletion inhibits ovarian cancer cell adhesion and migration through autophagy-mediated inhibition of RhoA

**DOI:** 10.1186/s13048-021-00767-3

**Published:** 2021-01-11

**Authors:** Nour El-Mais, Isabelle Fakhoury, Sandra Abdellatef, Ralph Abi-Habib, Mirvat El-Sibai

**Affiliations:** Department of Natural Sciences, School of Arts and Sciences, Lebanese American University, P.O. Box: 13-5053, Chouran, Beirut, 1102 2801 Lebanon

**Keywords:** Arginine deprivation, RhoA, Autophagy, Ovarian cancer, Cell motility

## Abstract

Ovarian carcinoma is the second most common malignancy of the female reproductive system and the leading cause of death from female reproductive system malignancies. Cancer cells have increased proliferation rate and thus require high amounts of amino acids, including arginine. L-arginine is a non-essential amino acid synthesized from L-citrulline by the Arginosuccinate synthetase (ASS1) enzyme. We have previously shown that the ovarian cancer cells, SKOV3, are auxotrophic to arginine, and that arginine deprivation by treatment with the genetically engineered human arginase I (HuArgI (Co)-PEG5000) triggers the death of SKOV3 cells by autophagy. In this study we examine the effect of HuArgI (Co)-PEG5000 on ovarian cancer cell migration and we dissect the mechanism involved. Wound healing assays, 2D random cell migration assays and cell adhesion analysis indicate that arginine deprivation decreases SKOV3 cell migration and adhesion. This effect was mimicked when autophagy was induced through rapamycin and reversed with the autophagy inhibitor chloroquine when autophagy was inhibited. This proved that arginine deprivation leads to the inhibition of cancer cell migration through autophagy, in addition to cell death. In addition, we were able to establish through pull-down assays and reversal experiments, that arginine deprivation-mediated autophagy inhibits cell migration through a direct inhibition of RhoA, member of the Rho family of GTPases. In conclusion, here we identify, for the first time, an autophagy-mediated inhibition of RhoA that plays an important role in regulating ovarian cancer cells motility and adhesion in response to arginine depletion.

**Supplementary Information:**

The online version contains supplementary material available at 10.1186/s13048-021-00767-3.

## Introduction

Ovarian cancer is the second most common malignancy of the female reproductive system and the leading cause of death from female reproductive system malignancies [1]. Drug toxicity, late diagnoses and metastasis remain some of the main challenges for treating ovarian carcinoma [2–4]. Hence there is an urgent need for new targeted anticancer agents with anti-migratory and anti-invasive potential.

Unlike normal cells, cancer cells have an increased proliferation rate and require high amounts of amino acids, including the non-essential amino acid arginine [5–7]. Arginine deprivation has recently emerged as a new approach for targeting cancer cells [6, 8]. Our previous studies uncovered a number of tumor models including ovarian cancer which exhibit complete or partial auxotrophy to arginine [9–13]. In addition our data demonstrated that arginine deprivation using pegylated human recombinant Arginase I cobalt [HuArgI (Co)-PEG5000] is selectively cytotoxic to cancer cells, namely, to acute lymphoid leukemia (ALL), hepatocellular carcinoma, glioblastoma multiforme (GBM), acute myeloid leukemia (AML), pancreatic cancer, colorectal cancer and ovarian cancer [9–13]. Our findings also revealed that arginine deprivation efficiently induces cell death by autophagy. Little however is known about the downstream signaling involved in arginine deprivation or its effects on cancer hallmarks, including the modulation of metastasis.

Cancer metastasis is the migration of tumor cells from the primary tumor site to a secondary one [14, 15]. Cell motility and adhesion regulation as well as actin cytoskeleton remodeling are required for cell metastasis and invasion to occur [16–19]. Mechanistically, moving cells dissolve cytoplasmic protrusions at the rear end of the cells but promote these structures at the leading edge along with stabilizing the cells to the substrate at focal adhesions [20, 21]. The Rho family of GTPases are the main regulators of actin cytoskeleton dynamics and cell motility and have been associated with pro-tumorigenic functions [22–28]. In addition, we have demonstrated the implication of RhoA of the family of Rho GTPases in colorectal cancer cells response to treatment with [HuArgI (Co)-PEG5000] [9].

Therefore, this study aimed at: 1- investigating the mechanism of action involved in SKOV3 cells response to treatment with HuArgI (Co)-PEG5000, 2- determining how arginine depletion affects cell migration, and adhesion, as well as 3- identifying key molecular targets mediating SKOV3 cell response to arginine deprivation.

## Materials and methods

### Reagents

Pegylated human recombinant Arginase I cobalt [HuArgI (Co)-PEG5000] (Pegzilarginase) was a gift from Aeaglea BioTherapeutics (Texas, USA). The constitutively active RhoA (CA-RhoA) and the empty vector plasmid (pcDNA3.1) constructs were also gifts from Dr. Yamaguchi Hideki. Chloroquine, rapamycin and L-citrulline were purchased from Sigma-Aldrich (Darmstadt, Germany). Cyto-ID autophagy detection kit was obtained from Enzo Life Sciences (New York, USA). RhoA/Rac1/Cdc42 Activation Assay Combo Kit was from Cell BioLabs (Sand Diego, CA, USA). Actin and vinculin primary antibodies were purchased from Abcam (Cambridge, UK). Rabbit polyclonal anti-LC3 antibody was obtained from Cell Signaling (Cell Signaling Technology Inc., US). Fluorescent secondary Alexa Fluor 488-green as well as Rhodamine phalloidin stain were obtained from Invitrogen (Massachusetts, USA). Hiperfect transfection reagent, luciferase GL2 and human Flexi Tubes siRNA for RhoA were bought from Qiagen (Hilden, Germany). Lipofectamine LTX was from Waltham (Massachusetts, USA) and crystal violet was from SCP Science (Quebec, Canada).

### Cell culture

SKOV-3 and Caov-3 ovarian adenocarcinoma cancer cell lines were purchased from ATCC (American Type Culture Collection). DMEM media supplemented with 10% fetal bovine serum, and 100 U penicillin/streptomycin was used to culture the cells in a humidified incubator at 37 °C and 5% CO_2_.

### Treatment

In all the experiments in this study, HuArgI (Co)-PEG5000 was used at a final concentration of 100 pM which approximately corresponds to its IC_50_ of the cells at 72 h post-treatment. For dose response experiments, cells were treated with IC_50_/3, IC_50_/2, IC_50_ or 10^− 8^ M or left untreated as indicated. The final concentrations used for rapamycin, L-citrulline and chloroquine were 0.5 μM, 11.4 mM, and, 10 μM, respectively.

### Transfection with siRNA and plasmid constructs

SKOV3 ovarian cancer cells were transfected with predesigned siRNA directed against human RhoA at a final concentration of 10 nM using the Hiperfect transfection reagent as per the manufacturer’s recommendations. Control cells were transfected with siRNA sequences targeting the GL2 Luciferase. Western blot analysis was performed to determine the efficiency of the knock down. Alternatively, SKOV3 cells were transfected with 5 μg of Rho-CA, or control empty vector (pcDNA3.1) using lipofectamine following the manufacturer’s instructions. All assays were performed 72 h following transfection with siRNA, or 48 h following transfection with the constructs.

### Western blot

Whole-cell lysates were obtained by scraping the cells with laemmli sample buffer containing 4% SDS, 20% glycerol, 10% β-mercaptoethanol, 0.004% bromophenol blue, and 0.125 M Tris HCl (pH 6.8). The proteins were separated by SDS-PAGE under standard conditions, before blotting onto a PVDF membrane as previously described [29–32]. After blocking with 5% bovine serum albumin for 1 h, the membranes were incubated overnight at 4 °C with the diluted primary antibodies (RhoA: 1:200 or actin 1:2500). Following, the membranes were washed and incubated with the appropriate secondary antibody (1:1000), for 1 h at room temperature before visualization using a chemiluminescent reagent. The images were captured using the Chemidoc imaging system from Biorad (California, USA). Protein expression levels were measured by densitometry analysis of the developed bands using ImageJ software (National Institute of Health, Massachusetts, USA).

### Pull down assay

SKOV3 cells were treated as indicated before extracting the proteins using the cell lysis buffer provided with the RhoA/Rac1/Cdc42 Activation Assay Combo Kit. Five hundred microliter of the cell lysates were then mixed with the GST-RBD beads and placed on a shaker at 4 °C for 1 h. After incubation, the samples were centrifuged, and the pellet was washed several times before resuspension in laemelli sample (referred to as GTP-RhoA samples). Five hundred microliter of the cell lysates collected prior to the incubation with GST-RBD were also mixed with laemelli buffer and used as a loading control which we refer to as total RhoA. Both total RhoA and GTP-RhoA were boiled for 5 min at 100 °C before separation by SDS-PAGE. The proteins were then transferred onto PVDF membranes and blocked with 5% BSA blocking solution for 1 h at room temperature. GTP-RhoA and total RhoA samples were detected by western blot using the anti-RhoA antibody provided with the kit. The levels of protein expression were quantified by densitometry analysis in ImageJ, and the data was presented as fold change relative to the control.

### Wound healing assay

SKOV3 cells were grown to confluence, treated as indicated then scratched with a sterile pipette tip to make a wound in the monolayer. Cell debris were washed and fresh medium was replenished. Phase-contrast images of the same wound area were taken directly after the wound was performed, and after 72 h using the 10X objective of a Leica inverted microscope. Wound widths were measured at 11 different positions for each sample, and the average rate of wound closure was calculated in μm/h using the ImageJ software.

### Random cell motility assay (time-lapse)

SKOV3 cells were treated as indicated and placed on a heated stage (37 °C) in a controlled CO_2_ environment (5%). Images of cells randomly moving in their respective media were collected every minute for 2 h using a 20X objective lens on the Zeiss Observer Z1 microscope. The total distance traveled by the cells was quantified using the ROI tracker plugin in ImageJ. The speed (μm/min) of at least 10 randomly selected cells per condition was then calculated by dividing the total distance traveled over time. Finally, the difference in cell motility was expressed as fold change of the treated SKOV3 cells normalized to the control.

### Adhesion assay

For cell adhesion assay, 96-well plates were coated with collagen type I overnight at 37 °C. The next day, the plates were washed with washing buffer (0.1% BSA in DMEM media) and blocked in 0.5% BSA blocking solution at 37 °C for 1 h. Next, 50 μl of SKOV3 cell suspension (density = 4 × 10^5^ cells/ml) were plated in the coated wells, and incubated at 37 °C and 5% CO_2_ for 30 min. Following, the media was removed and the wells were washed 3 times before fixing with 4% paraformaldehyde at room temperature for 10 min. The adherent cells in the wells were then stained with crystal violet (5 mg/ml in 2% ethanol) for 10 min. Finally, the plates were thoroughly washed with water and dried before solubilizing crystal violet in 2% SDS for 30 min. Colorimetric quantification of the dye was performed at 550 nm using an ELISA plate reader.

### Invasion assay

Invasion assay was performed as previously described using the collagen-based invasion assay kit from Millipore (Burlington, MA) [9]. Briefly, control and transfected ovarian cancer cells were starved for 24 h before resuspension in serum-free quenching medium and plating onto the hydrated inserts. The cells were then placed in wells containing complete medium (10% FBS) and incubated for 24 h. Following, the cells at the bottom surface of the inserts were stained with 400 μl of cell stain for 20 min at room temperature. After extracting the stain with the extraction buffer, 100 μl of the extracted stain were transferred to the wells of a 96-well plate. Finally, the optical density of each sample was measured at 560 nm using the Varioskan microplate reader from ThermoFisher scientific (Massachusetts, USA).

### Immunofluorescence

For immunostaining experiments, SKOV3 ovarian cancer cells were plated on glass coverslips and treated as indicated for 72 h. Next, the cells were fixed with 4% paraformaldehyde for 10 min at 37 °C before permeabilizing with 0.5% Triton-X 100 for 15 min on ice. Following, the samples were blocked in 1% BSA solution for 1 h, incubated with the primary antibodies overnight at 4 °C, and subsequently with the green fluorophore-conjugated secondary antibodies for 1 h. Fluorescent images were taken using a 63X objective lens on Zeiss Observer Z1 microscope operated by the Zen software (Zeiss, Oberkochen, Germany).

### Autophagy assay

Autophagy was assessed using the Cyto-ID autophagosome detection kit following the manufacturer’s instructions. Briefly, 2 × 10^4^ cells/ml were plated on glass coverslips and treated as indicated for 72 h. The media was removed, and the cells were washed several times before incubation with the Cyto-ID stain for 40 min. Next, the cells were washed, fixed with paraformaldehyde for 10 min at 37 °C and permeabilized with 0.5% Triton-X 100 for 15 min on ice. Finally, Fluorescent images were taken using a 63X objective lens on Zeiss Observer Z1 microscope operated by the Zen software (Zeiss, Oberkochen, Germany).

### Quantification of focal adhesions

CLAHE and Log3D plugins were used to quantify the area and numbers of focal adhesions in ImageJ (National Institute of Health, MA, USA) as describe previously [9, 33]. CLAHE enhances the local contrast of the image and Log3D filters the image based on predefined parameters for focal adhesions detection and analysis [9]. The area of focal adhesions observed following vinculin staining was expressed in arbitrary unit (a.u.). The number of focal adhesions was expressed as absolute value of the means for each condition.

### Statistical analysis

The data is representative of at least three independent experiments. The results are expressed as the mean ± SEM. The *p*-values were calculated using t-test and the statistical significance was set at *p*-value ≤0.05.

## Results

### Arginine depletion inhibits ovarian cancer cell motility

**Fig. 1:**
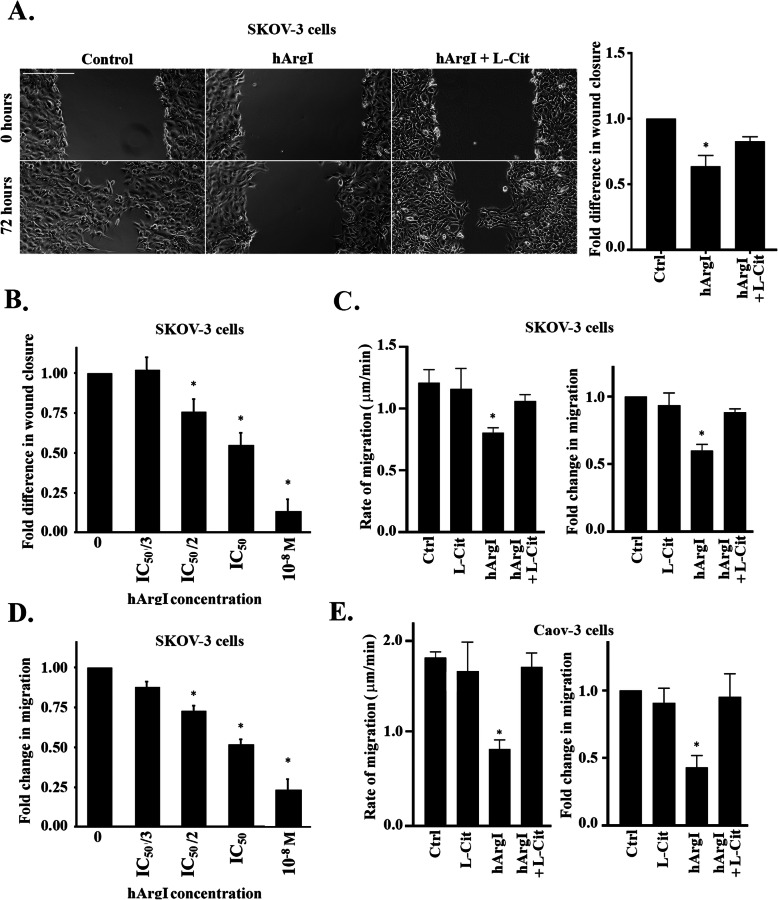
Arginine depletion inhibits ovarian cancer cell motility. **a** Control, hArgI treated and SKOV3 cells treated with hArgI in combination with citrulline were grown to confluency before creating a wound in the cell monolayer. Cell images of the same wound frame area were taken directly after the wound was made (t = 0) and 72 h later (t = 72). The micrographs represent the wound profile of the cells treated as indicated at t = 0 and t = 72. Scale bar is 100 μm. The graph is a quantitation of the wound closure rate using ImageJ. The wound closure rate is expressed as fold change relative to the untreated control. The data represent the mean ± SEM from 3 assays. The results were significant with *p* < 0.05. **b** Dose response in wound closure rate of SKOV3 cells treated with the indicated increasing concentrations of hArgI. Data are expressed relative to the untreated control and represent the mean ± SEM from 3 assays. The results were significant with *p* < 0.05. **c** SKOV3 cells were treated as described previously for 72 h before images of randomly moving cells in media were captured. Cell images were captured at 1 min interval for 2 h. The average migrated distance (in μm) was measured by tracking the images using ImageJ software and the speed was calculated by dividing the distance over time. Left panel: Bar graph illustrating the speed (μm/min) of control, hArgI treated, and hArgI treated SKOV3 cells in combination with citrulline, respectively. Right panel: Bar graph illustrating the fold change of hArgI treated SKOV3 cells migration relative to the control. **c** Dose response in random migration of SKOV3 cells treated with the indicated increasing concentrations of hArgI. Cell images were captured at 1 min interval for 2 h. The average migrated distance (in μm) was measured by tracking the images using ImageJ software and the speed was calculated by dividing the distance over time. Data are expressed as fold change of the migration of SKOV3 treated with different concentrations of hArgI relative to the untreated control. **e** Caov-3 cells were treated as described previously for 72 h before images of randomly moving cells in media were captured. Cell images were captured at 1 min interval for 2 h. The average migrated distance (in μm) was measured by tracking the images using ImageJ software and the speed was calculated by dividing the distance over time. Left panel: Bar graph illustrating the speed (μm/min) of control, hArgI treated, and hArgI treated SKOV3 cells in combination with citrulline, respectively. Right panel: Bar graph illustrating the fold change of hArgI treated SKOV3 cells migration relative to the control

We previously studied the effect of arginine deprivation on the proliferative ability of ovarian cancer cells [13]. Having also proven that arginine depletion halted cancer cell migration in other tumor types [9], we investigated the effect of HuArgI (Co)-PEG5000 treatment on the motility of the high-grade serous (HGS) ovarian adenocarcinoma cells, SKOV3 and Caov-3. This was performed using two approaches: wound healing, and time-lapse assays. Treating SKOV3 cells with 100 pM HuArgI (Co)-PEG5000 decreased the rate of wound closure by 0.4 fold compared to the control (Fig. 1a). This effect was reversed when the cells were treated with HuArgI (Co)-PEG5000 in combination with L-citrulline (11.4 mM), thus proving the role of arginine depletion in modulating cancer cell motility. Time lapse assay also confirmed these observations whereby treatment of SKOV3 cells with HuArgI (Co)-PEG5000 alone or in combination with L-citrulline, respectively, decreased cell motility and reversed this decrease (Fig. 1c, Supplemental movie S1 and S2), while treatment with L-citrulline alone had no effect on cell motility. Specifically, the data shows that the speed of migration of control SKOV3 cells is significantly reduced upon treatment with HuArgI (Co)-PEG5000 (Fig. 1c). In comparison, arginine depletion in combination with treatment with L-citrulline reversed the speed of migration to almost control levels. A dose response was also performed, treating SKOV3 cells with escalating doses of the arginase during the wound healing or the time lapse experiment and showed an increasing inhibition with the increased dose of treatment, reflecting specificity of the effect (Fig. 1b and d and Supplemental movie S3). The same effect was also seen in Caov-3 cells with an even more pronounced inhibition of random cell migration by arginine deprivation and a complete reversal of the effect upon parallel treatment with L-citrulline (Fig. 1e and Supplemental movie S4). The dose response was also similar in Caov-3 cells with movie only showing the complete cell death upon treating Caov-3 cells with 10^− 8^ M hArgI (Supplemental movie S4). Contrary to observations from other tumor types [9], arginine deprivation did not have an effect on the invasion of SKOV3 cells or Caov-3 cells (Supplemental Figure S1A and S1B, respectively), hence we set out to solely examine ovarian cell migration and to elucidate the mechanism of inhibition by arginine deprivation in these cells.

### Arginine depletion decreases ovarian cancer cell adhesion to collagen

**Fig. 2:**
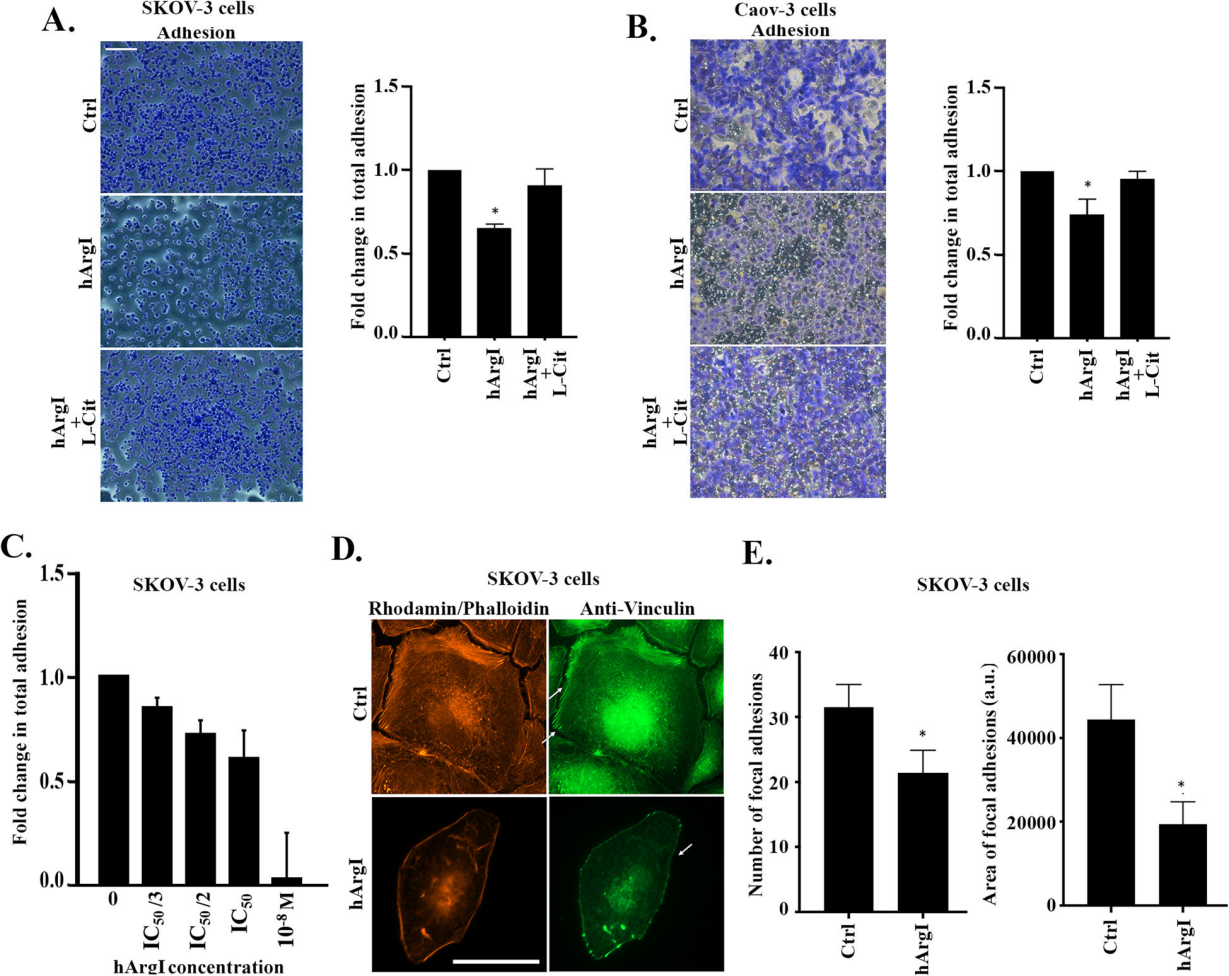
Arginine depletion decreases ovarian cancer cell adhesion to collagen. **a** Representative micrographs of control, hArgI treated and hArgI + citrulline treated SKOV3 cells after fixation and staining with crystal violet to detect adhesion. Scale bar is 100 μm. Colorimetric quantification of the dissolved dye was performed at 560 nm using an Elisa plate reader. The bar graph represents the quantification of adherent cells expressed as fold increase relative to the untreated control. Significance was set at *p* < 0.05. **b** Representative micrographs of control, hArgI treated and hArgI + citrulline treated Caov-3 cells after fixation and staining with crystal violet to detect adhesion. Scale bar is 100 μm. Colorimetric quantification of the dissolved dye was performed at 560 nm using an Elisa plate reader. The bar graph represents the quantification of adherent cells expressed as fold increase relative to the untreated control. Significance was set at *p* < 0.05. **c** Dose adhesion response of SKOV3 cells to the indicated concentrations of hArgI. Data expressed as fold change relative to the untreated control. Significance was set at *p* < 0.05. **d** Control and hArgI treated cells were immunostained with vinculin (green) and Rhodamine phalloidin (orange). SKOV3 treated cells were imaged using a 63x objective of a fluorescent microscope. Scale bar = 10 μm. **e** Bar graphs illustrating the quantification of the area of focal adhesions (right panel) and the number of focal adhesions (left panel) were evidenced by vinculin staining and generated using the CLAHE and Log3D plugins in ImageJ. The area of focal adhesion is expressed in arbitrary unit (a.u.) and the number of focal adhesion is shown as absolute values of the means in every condition

To understand how arginine deprivation inhibits cell motility in 2D, we assessed the effect of the treatment on the adhesion of SKOV3 cells to collagen; a main component of the extracellular matrix (ECM). Figure 2a and b reveal that treatment with HuArgI (Co)-PEG5000 decreases cell adhesion as compared to the control, and that the combination of HuArgI (Co)-PEG5000 with L-citrulline reverses this decrease. Quantitatively, HuArgI (Co)-PEG5000 reduced cell adhesion by 0.35 fold in SKOV3 cells and around 0.3 fold in Caov-3 cells and the addition of L-citrulline reversed this decrease to almost control levels in both cells (Fig. 2a and b). A dose response was performed, treating SKOV3 cells with escalating doses of the arginase while allowing the cells to adhere. This showed an increasing inhibition of adhesion with the increased dose of treatment with an almost complete lack of adhesion at 10^− 8^ M hArgI treatment, reflecting specificity of the effect (Fig. 2c). Immunostaining the cells with an actin specific stain as well as a marker for actin-rich focal adhesions further supported this finding. Indeed, staining with anti-vinculin and Rhodamine phalloidin shows that the depletion of arginine in SKOV3 results in the loss of cell definition, and contractility (Fig. 2d). The cells also appear more flat and extended in all directions with complete absence of actin stress fibers (Fig. 2d). This was finally translated in a decrease in the area and the number of focal adhesions by 10, and 54%, respectively, in cells treated with HuArgI (Co)-PEG5000 as compared to control (Fig. 2e).

### Arginine depletion decreases cell adhesion by inhibiting RhoA in ovarian cancer cells

**Fig. 3:**
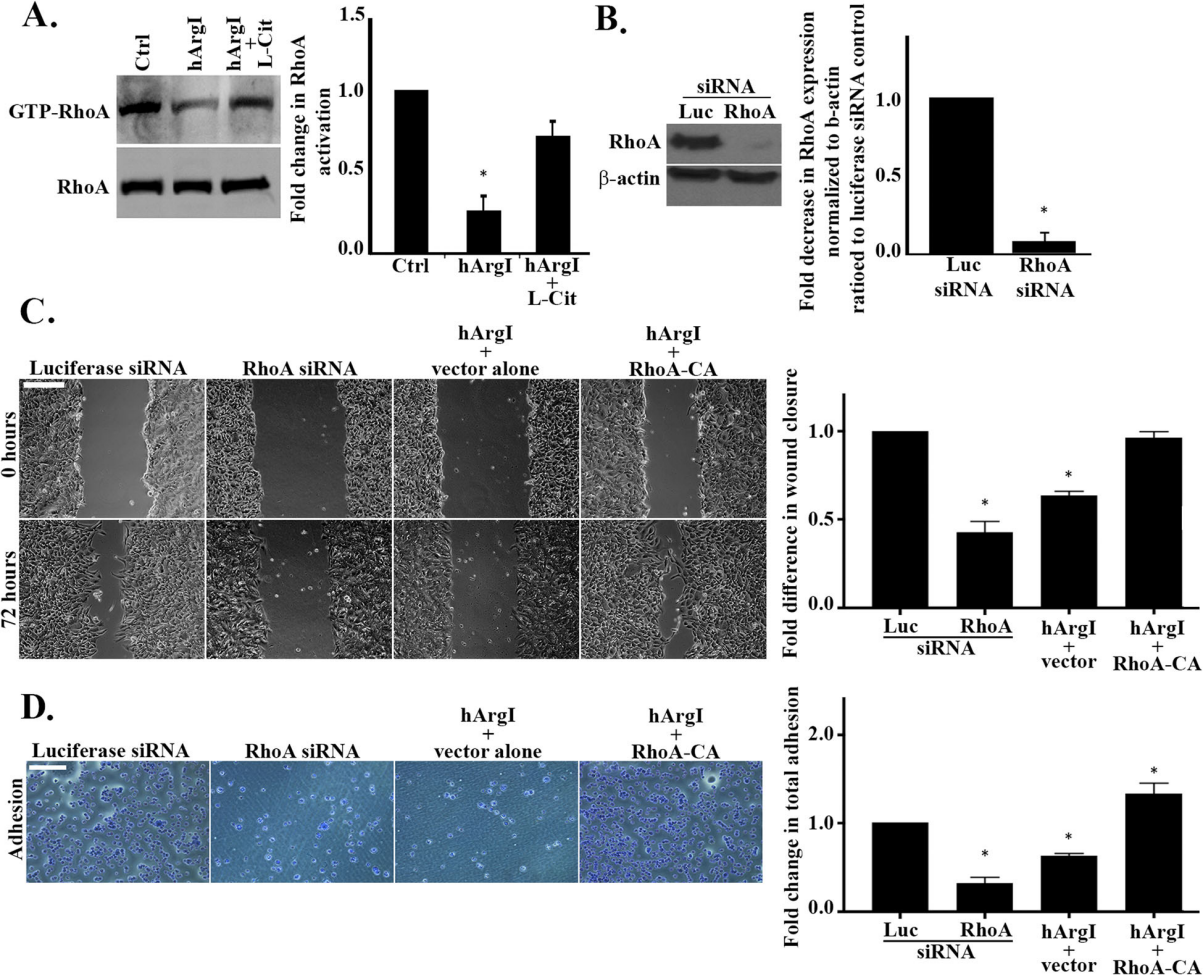
Arginine depletion decreases cell adhesion by inhibiting RhoA in SKOV3 cells. **a** Control and hArgI or hArgI + citrulline treated SKOV3 cell extracts were incubated with GST-RBD beads before blotting against RhoA. Total cell lysates which were not incubated with the beads were also blotted against RhoA and used as loading control. Right panel: Bar graph illustrating the amount of RhoA activation expressed as fold change of the activated RhoA normalized to the amount of total protein. The densitometry quantification of active and total RhoA bands was performed in ImageJ. **b** SKOV3 ovarian cancer cells were transfected with Luciferase siRNA or RhoA siRNA for 72 h before protein extraction and blotting against RhoA. Left panel: Western blot profile showing the expression levels of RhoA and beta actin in SKOV3 cells. Right panel: Quantification of RhoA expression levels by ImageJ. The data is presented as fold decrease in RhoA expression levels normalized to beta actin of the siRNA RhoA transfected cells relative to the luciferase control. Data are the mean ± SEM from 3 assays. The results were significant with *p* < 0.05. **c** SKOV3 cells were transfected with Luciferase siRNA, RhoA siRNA, or treated with hArgI and transfected with an empty vector or a constitutively active RhoA construct, respectively. All samples were grown to confluency before creating a wound in the cell monolayer and imaging the wound area at (t = 0) and 72 h later (t = 72). The micrographs represent the wound profile of the same area at the indicated time points. Scale bar is 100 μm. Right panel: Bar graph illustrating the quantification of the wound closure rate expressed as fold changes relative to the luciferase control. The results were significant with *p* < 0.05. **d** Left panel: Representative micrographs of fixed and stained SKOV3 cells which were transfected with Luciferase siRNA, RhoA siRNA, or treated with hArgI and transfected with an empty vector or the constitutively active RhoA construct, respectively. Scale bar is 100 μm. Right panel: Colorimetric quantification of the dissolved crystal violet stain was performed at 560 nm using an Elisa plate reader. The bar graph represents the quantification of adherent cells expressed as fold increase relative to the luciferase control. Significance was set at *p* < 0.05

The cell phenotype observed after treatment with HuArgI (Co)-PEG5000, along with the decrease in cell adhesion, particularly the decrease in the area of adhesion structures, suggested a potential decrease in RhoA activation in response to arginase treatment, similar to what we have previously observed in colon cancer [9]. Therefore we investigated RhoA activation in SKOV3 cells. Pull down analysis shows that treatment of SKOV3 cells with HuArgI (Co)-PEG5000 inactivates RhoA by around 0.75 fold as compared to the control (Fig. 3a). On the contrary, treatment with HuArgI (Co)-PEG5000 in combination with L-citrulline restores RhoA activation by 0.5 fold. To further prove the role of arginine deprivation in the decrease in activation of RhoA, we depleted RhoA from SKOV3 ovarian cancer cells (Fig. 3b). This was achieved by transfection of the cells with RhoA specific siRNA, or a nonspecific negative control (Luciferase siRNA). Western blot analysis demonstrated that the oligos used efficiently inhibited the expression of RhoA. Specifically, RhoA expression levels were reduced by 0.9 fold in si-RhoA transfected SKOV3 cells as compared to the control (Luciferase siRNA). Moreover, our data indicates that knocking down RhoA mimics arginine deprivation and decreases the rate of wound closure by 0.6 fold compared to the Luciferase siRNA control. Consistently, transfection of a constitutively active RhoA countered HuArgI (Co)-PEG5000 treatment and restored the rate of wound closure to that of control levels (Fig. 3c). RhoA knock down had similar effects on the adhesion of SKOV3 cells. Indeed, Fig. 3d illustrates that knocking down RhoA decreases cell adhesion (by 0.65 fold) in a way that is similar to that of arginine deprivation from SKOV3 cells (0.5 fold). Moreover, the transfection of a constitutively active RhoA countered HuArgI (Co)-PEG5000 treatment and completely restored cell adhesion to control levels (Fig. 3d).

### Arginine depletion-mediated decrease in cell adhesion and motility is through the stimulation of autophagy in ovarian cancer cells

**Fig. 4:**
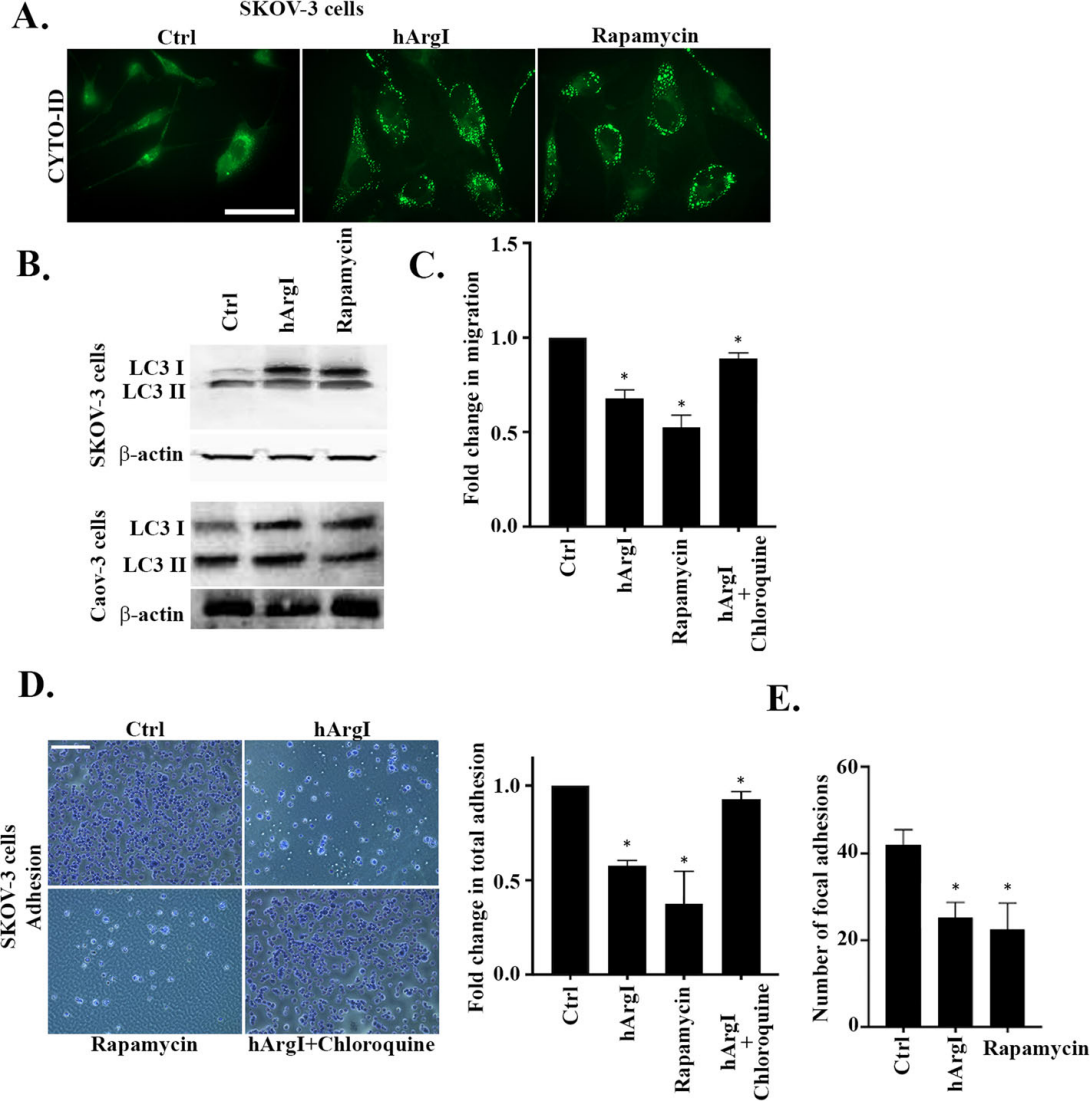
Arginine depletion-mediated decrease in cell adhesion and motility is through the stimulation of autophagy in ovarian cancer cells. **a** Left panel: Micrographs illustrating SKOV3 cells treated with hArgI or rapamycin and stained with Cyto-ID (green). Fluorescent images were taken using the 63x objective lens of the Zeiss Observer Z1 fluorescent microscope. Scale bar is 10 μm. **b** LC3 cleavage in SKOV3 (upper panel) and Caov-3 (lower panel) and actin blot for loading control (lower gels) following treatment with hArgI or rapamycin on western blot. **c** SKOV3 cells were treated with hArgI, rapamycin or hArgI in combination with chloroquine before being imaged moving randomly in media. Cell images were captured at 1 min interval for 2 h.The average migrated distance (in μm) was measured by tracking the images using ImageJ software and the speed was calculated by dividing the distance over time. The bar graph illustrates the fold change in migration of hArgI, rapamycin or hArgI + chloroquine treated SKOV3 cells normalized to the untreated control. **d** Representative micrographs of fixed and stained control SKOV3 cells, and SKOV3 cells treated with either hArgI, rapamycin or hArgI in combination with chloroquine. Scale bar is 100 μm. Right panel: Colorimetric quantification of the dissolved crystal violet stain of the cells described in C was performed at 560 nm using an Elisa plate reader. The bar graph represents the quantification of adherent cells expressed as fold increase relative to the untreated control. Significance was set at *p* < 0.05. **e** Bar graph illustrating the number of focal adhesions in control and hArgI or rapamycin treated cells stained with anti-vinculin. The number of focal adhesion was quantified using the CLAHE and Log3D plugins in Image and is expressed as absolute values of the means for every sample

Previous work in our lab demonstrated that arginine deprivation triggers autophagy [10, 13]. We and other have also established a role for autophagy in the regulation of cell migration [9, 34–37]. Therefore we investigated the role of autophagy in arginine deprivation-mediated regulation of cell migration and adhesion of ovarian cancer cells. Figure 4a reveals a clear activation of autophagy through CytoID analysis in SKOV3 cells upon arginine deprivation comparable to that observed upon treatment with rapamycin, a known positive activator of autophagy. We also observe an increase in the level of cleaved microtubule-associated proteins 1A/1B light chain 3B (LC3) protein, LC3 I (which reflects activated autophagy), in SKOV3 and Caov-3 cells in response to both hArgI and rapamycin treatment (Fig. 4b). Using chloroquine, the inhibitor of autophagy, we further proved that the autophagy regulates the cell motility of arginine depleted SKOV3 cells by autophagy. Indeed, the random cell motility data demonstrated that rapamycin, and arginine deprivation decrease cell motility by 0.5 and 0.35 fold, respectively, as compared to the control (Fig. 4c and Supplemental movie S5). In contrast, treatment of SKOV3 cells with HuArgI (Co)-PEG5000 in combination with chloroquine restored cell motility to control levels. Autophagy was also required for SKOV3 mediated decrease in cell adhesion following arginine deprivation. The data shown in Fig. 4d indicates that both rapamycin and arginine deprivation decrease cell adhesion, by 0.6 And 0.45 folds, respectively, and that chloroquine treatment in combination with HuArgI (Co)-PEG5000 restores cell adhesion to control levels. This was further corroborated by the number of focal adhesions quantified following SKOV3 cells treatment with rapamycin or HuArgI (Co)-PEG5000. Both conditions, reduced the number of focal adhesions as compared to the control by approximately 0.5 fold (Fig. 4e). Altogether, this demonstrates that the effect of arginine deprivation on the migration and adhesion of SKOV3 cells is mediated by autophagy.

### Autophagy decreases cell motility by inhibiting RhoA in ovarian cancer cells

**Fig. 5:**
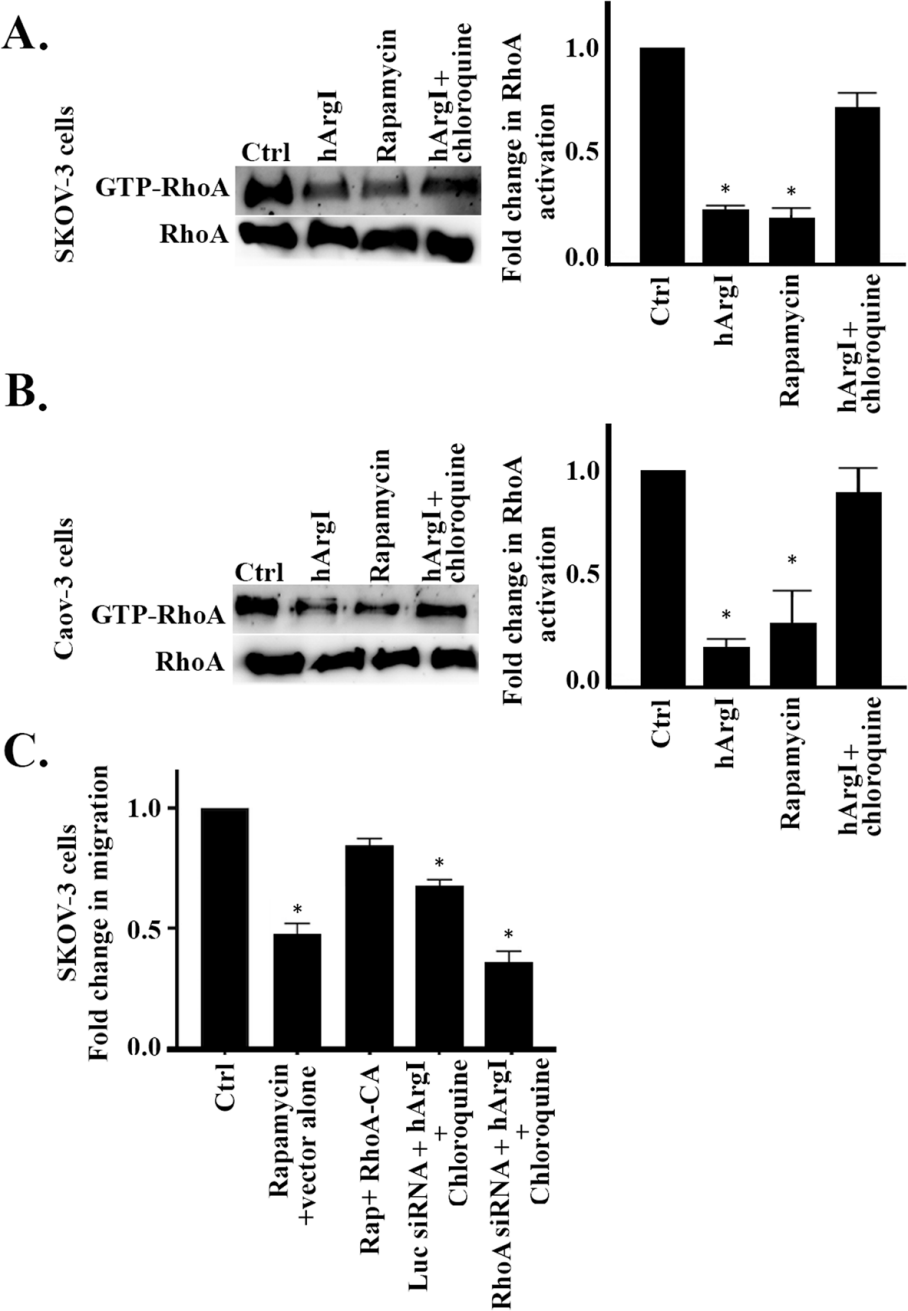
Autophagy decreases cell motility by inhibiting RhoA in ovarian cancer cells. **a** SKOV3 cells treated with hArgI, rapamycin or hArgI + chloroquine or left untreated. SKOV3 cell extracts were incubated with GST-RBD beads before blotting against RhoA. Total cell lysates which were not incubated with the beads were also blotted against RhoA and used as loading control. Right panel: Bar graph illustrating the amount of RhoA activation expressed as fold change of the activated RhoA normalized to the amount of total protein. The densitometry quantification of active and total RhoA bands was performed in ImageJ. **b** Caov-3 cells treated with hArgI, rapamycin or hArgI + chloroquine or left untreated. Caov-3 cell extracts were incubated with GST-RBD beads before blotting against RhoA. Total cell lysates which were not incubated with the beads were also blotted against RhoA and used as loading control. Right panel: Bar graph illustrating the amount of RhoA activation expressed as fold change of the activated RhoA normalized to the amount of total protein. The densitometry quantification of active and total RhoA bands was performed in ImageJ. **c** SKOV3 cells were untreated or treated as indicated with Rapamycin, Rapamycin+RhoA-CA, Luciferase siRNA+hArgI+Chloroquine or RhoA siNRA + hArgI+Chloroquine before being imaged moving randomly in media. The bar graph illustrates the fold change of treated SKOV3 cells migration relative to the control

Next we investigated the regulation of RhoA activation in response to autophagy in SKOV3 and Caov-3 cells. Figure 5a and b shows that RhoA activation decreases by around 0.8 fold upon treatment with rapamycin and that treatment with the autophagy inhibitor chloroquine in combination with HuArgI (Co)-PEG5000 restores RhoA activation levels. The results confirm that autophagy reduces ovarian cancer cells motility by inhibiting RhoA (Fig. 5c and Supplemental movie S6). Specifically, the data shows that the motility of SKOV3 cells transfected with the empty vector and treated with rapamycin (rapamycin + vector alone) decreases by 0.5 fold as compared to the control. Transfection of SKOV3 cells with the constitutively active RhoA construct counters the effect of rapamycin and restores cell motility to that of control levels (rapamycin + RhoA-CA). Furthermore, we showed that chloroquine only counters the effects of arginine deprivation in SKOV3 cells expressing RhoA (Luc siRNA + hArgI + chloroquine) and not in RhoA depleted cells (RhoA siRNA + hArgI + chloroquine) which exhibited a cell motility decrease of about 0.6 fold.

## Discussion

The cytotoxic effects of arginine deprivation have been widely investigated in various tumor models [5, 10–12, 38, 39]. However, arginine deprivation mechanisms of action in cancer in general, and in ovarian carcinoma specifically, remain poorly understood. Therefore, we performed this study to understand HuArgI (Co)-PEG5000 effects on cell motility, and identify key molecular targets which mediate cancer cells response to treatment with arginase.

Using 2D motility assays, we proved that arginase decreases cell migration of SKOV3 and Caov-3 cancer cell line. This is in line with previous work performed in our lab on colorectal cancer cells which revealed arginase’s ability to modulate different cancer hallmarks including proliferation, cell motility and invasion [9]. This effect has also been reported by other research groups in pancreatic cancer and in glioblastoma models [40, 41]. Surprisingly here, arginase did not modulate the invasion of ovarian cancer cells, thus suggesting a possible tumor specific response to treatment with HuArgI (Co)-PEG5000 [9]. In addition, often 2D migration and invasion seem to be regulated by different mechanisms in mammalian cells [29].

Indeed, 2D cell motility dynamics involve the formation of cell protrusions and the dissolution of adhesive structures [17, 42]; therefore we investigated the drug’s effects on these components. Our data indicate a decrease in the stabilization and the adhesion of ovarian cancer cells to the ECM, as well as a decrease in the number of focal adhesions. This decrease in adhesion is contradictory with the reduced cell motility findings discussed earlier. However, the distinctive loss of cell definition, contractility and directionality, along with the increase in cell extension and flatness suggested a potential implication of the RhoA Rho GTPase similar to that observed in colon cancer in response to arginine deprivation [9]. Moreover, the role of the RhoA Rho kinase pathway in the activation of biological arginase had been previously described in the literature in non-cancer models [43–47]. As predicted, this study confirmed that arginine deprivation decreases RhoA activation. This is in line with RhoA effects on the regulation of cell motility and morphology [48]. Interestingly, treatment of ovarian cancer cells with L-citrulline in combination with HuArgI (Co)-PEG5000 rescued the cells from arginine deprivation-induced decrease in cell motility, cell adhesion and RhoA inactivation. L-citrulline is a precursor of arginine as well as a marker of complete arginine auxotrophy. We have previously demonstrated the complete auxotrophy of SKOV3 cells to arginine. Specifically, the data showed that excess L-citrulline fails to rescue SKOV3 from arginine deprivation-induced cytotoxicity [13]. L-citrulline is thus capable of reversing arginine deprivation effects on SKOV3 and Caov-3 cell motility, cell adhesion and RhoA inhibition, but cannot reverse HuArgI (Co)-PEG5000 induced cytotoxicity.

Moreover, this works highlights the similarity between RhoA depletion and arginine deprivation in terms of regulation of the migration and the adhesion dynamics of ovarian cancer cells. The pheno-mimicking further suggests a role for RhoA in mediating arginine deprivation effects, which was confirmed upon transfection of cells with the constitutively active RhoA in combination with HuArgI (Co)-PEG5000. To our knowledge; this represents the first evidence proving that arginine deprivation requires RhoA inactivation to mediate its effects against cell motility and adhesion. It also highlights the need to test this hypothesis in our colon cancer cells model to determine whether RhoA contribution to arginine deprivation downstream effects is similar to that observed in ovarian cancer cells.

Previous work in our lab uncovered that arginine deprivation stimulates autophagy in various cancer models including pancreatic and ovarian cancers [10, 13]. We confirmed these findings using the CytoID stain and LC3 cleavage analysis, which revealed the formation of autophagosomes in ovarian cancer cells upon treatment with HuArgI (Co)-PEG5000. Studies in the literature have established a role for autophagy in the regulation of cell migration uncovering both cell migration promoting and inhibitory effects for autophagy [36, 49–51]. We thus investigated the role of autophagy in the regulation of cell migration and adhesion of ovarian cancer cells. This study demonstrates that autophagy stimulation mediates the decrease of cell migration and adhesion upon arginine depletion. The data further proved that RhoA inhibition is required for autophagy-mediated decrease in cell motility and adhesion. This is consistent with other reports which demonstrated that autophagy suppression of cell migration involves direct or indirect inhibition of RhoA [37, 52].

## Conclusion

This study demonstrates that arginase regulation of ovarian cancer cells motility and adhesion involves autophagy-mediated inhibition of RhoA. This uncovers the mechanism of action of arginine deprivation and highlights a key role for autophagy and the Rho GTPases in the regulation of cancer cells invasiveness. Altogether, arginase ability to modulate cell migration and key molecules involved in cancer malignancy makes it a great candidate for cancer therapeutics.

## Supplementary Information

**Additional file 1 **: **Supplemental Figure S1.** Arginine deprivation does not affect the invasion of ovarian cancer cellsSKOV3 cancer cells. SKOV3 (A) OR Caov-3 (B) cells were treated with or without hArgI and allowed to invade collagen-coated membranes towards 10%FBS. Quantification of the invaded cells was performed 24 h after cell stain extraction and presented as fold change of treated cells normalized to the control.

**Additional file 2 **: **Supplemental movie S1.** Time-lapse movie illustrating SKOV3 cells treated with; from left panel/movie to right panel: Control, L-Citrulline, hArgI, hArgI + L-Citrulline and undergoing random motility in serum. Time lapse movie is for 2 h with a frame taken every minute (phase contrast, 20X objective).

**Additional file 3 **: **Supplemental movie S2.** Movie showing the original full fames (20X objective) of the zoomed selected ROIs (regions of interests) from Supplemental movie S1.

**Additional file 4 **: **Supplemental movie S3.** Time-lapse movie illustrating SKOV3 cells treated with; from left panel/movie to right panel: Control, hArgI IC_50_/3, hArgI IC_50_ and 10^− 8^ M hArgI and undergoing random motility in serum. Time lapse movie is for 2 h with a frame taken every minute (Phase contrast, 20X objective).

**Additional file 5 **: **Supplemental movie S4.** Time-lapse movie illustrating Caov-3 cells treated with; from left panel/movie to right panel: Control, L-Citrulline, hArgI IC_50_, hArgI + L-Citrulline and 10^− 8^ M hArgI and undergoing random motility in serum. Time lapse movie is for 2 h with a frame taken every 2 min (Movies are zoomed and cropped, phase contrast, 20X objective).

**Additional file 6 **: **Supplemental movie S5.** Time-lapse movie illustrating SKOV3 cells treated with; from left panel/movie to right panel: Control, hArgI, Rapamycin and hArgI combined with Chloroquine and undergoing random motility in serum. Time lapse movie is for 2 h with a frame taken every minute (Phase contrast, 20X objective).

**Additional file 7 **: **Supplemental movie S6.** Time-lapse movie illustrating SKOV3 cells treated with; from left panel/movie to right panel: Control, Rapamycin + empty vector, Rapamycin + RhoA-CA, hArgI + Chloroquine + Luciferase siRNA and finally hArgI + Chloroquine + RhoA siRNA and undergoing random motility in serum. Time lapse movie is for 2 h with a frame taken every minute (Phase contrast, 20X objective).

## Data Availability

All data is available upon request with a reasonable time.
