# Supplementary figures and images for: Human recombinant arginase I [HuArgI (Co)-PEG5000]-induced arginine depletion inhibits ovarian cancer cell adhesion and migration through autophagy-mediated inhibition of RhoA
Source: J Ovarian Res. 2021 Jan 11;14:13. doi: 10.1186/s13048-021-00767-3 (PMC7798344; doi:10.1186/s13048-021-00767-3)

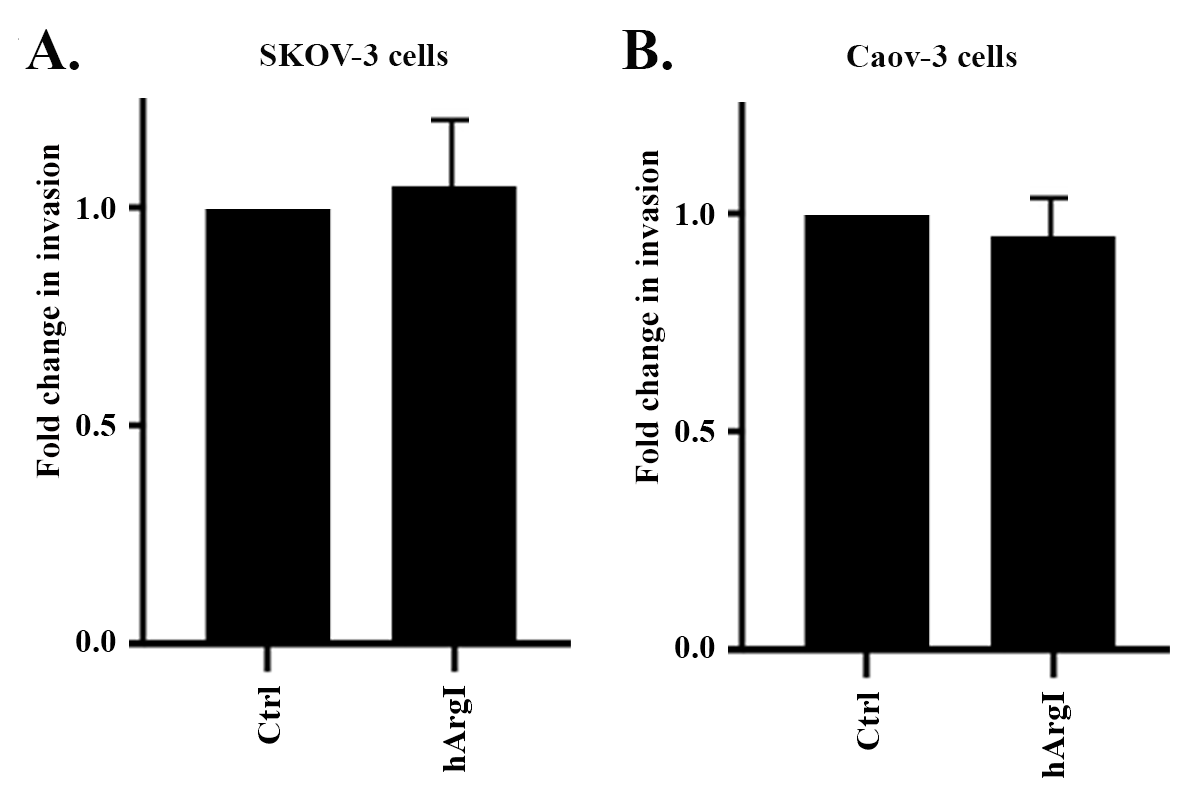

Supplement: Supplementary file 1 — Additional file 1 : Supplemental Figure S1. Arginine deprivation does not affect the invasion of ovarian cancer cellsSKOV3 cancer cells. SKOV3 (A) OR Caov-3 (B) cells were treated with or without hArgI and allowed to invade collagen-coated membranes towards 10%FBS. Quantification of the invaded cells was performed 24 h after cell stain extraction and presented as fold change of treated cells normalized to the control. [file 13048_2021_767_MOESM1_ESM.tif]
